# Supplementary material for: Estimating the dietary and health impact of implementing mandatory front-of-package nutrient disclosures in the US: A policy scenario modeling analysis
Source: PLoS One. 2025 Feb 24;20(2):e0312638. doi: 10.1371/journal.pone.0312638 (PMC11849812; doi:10.1371/journal.pone.0312638)
Supplement: S1 File — S1 Table: Sample characteristics, adults (≥19 year). S2 Table: Baseline and counterfactual scenarios: US adults’ (≥19 y) usual mean energy and targeted nutrient intakes by DRI age/sex group (n = 7,572). S3 Table: Diet related NCD deaths that could be averted or delayed in the US due to changes in calorie and nutrients-of-concern content in food and beverage purchases in the presence of nutrient-specific ‘high in’ FOPL. (DOCX) [file pone.0312638.s001.docx]

**Supporting information**

**Estimating the dietary and health impact of implementing mandatory** **front-of-package nutrient disclosures in the US: a policy scenario modeling analysis**

Nadia Flexner,^1,2^ Daniel Zaltz,^1^ Eva Greenthal,^3^ Aviva A. Musicus,^3,4^ Mavra Ahmed,^1,5^ Mary R. L’Abbe^1*^

^1^Department of Nutritional Sciences, Temerty Faculty of Medicine, University of Toronto, Toronto, ON, Canada.

^2^Global Health Advocacy Incubator, Washington, DC, USA.

^3^Center for Science in the Public Interest, Washington, DC, USA.

^4^Department of Nutrition, Harvard T.H. Chan School of Public Health, Boston, MA, USA.

^5^Joannah & Brian Lawson Centre for Child Nutrition, Temerty Faculty of Medicine, University of Toronto, Toronto, ON, Canada.

^*^Corresponding author

Email: [mary.labbe@utoronto.ca](mailto:mary.labbe@utoronto.ca) (MRL)

Table of Contents

[S1 Table. Sample characteristics, adults (≥19 year) ^*^ 2](#_Toc187616199)

[S2 Table. Baseline and counterfactual scenarios: US adults’ (≥19 y) usual mean energy and targeted nutrient intakes by DRI age/sex group (n=7,572) 3](#_Toc187616200)

[S3 Table. Diet related NCD deaths that could be averted or delayed in the US due to changes in calorie and nutrients-of-concern content in food and beverage purchases in the presence of nutrient-specific ‘high in’ FOPL. 9](#_Toc187616201)

## **S1 Table.** Sample characteristics, adults (≥19 year) ^*^

| **Characteristic** | n = 7,572*^1^* |
| --- | --- |
| **Sex, % (n)** |  |
| Female | 51% (3,842) |
| Male | 49% (3,730) |
| **BMI Category, % (n)** |  |
| Normal weight (18.5–24.9 kg/m^2^) | 25% (1,843) |
| Overweight (25.0–29.9 kg/m^2^) | 32% (2,376) |
| Obese (>30.0 kg/m^2^) | 44% (3,299) |
| **BMI by DRI age-sex category, mean (SD)** |  |
| Male, 19-30 | 28.2 (6.7) |
| Female, 19-30 | 29.5 (8.4) |
| Male, 31-50 | 30.4 (6.7) |
| Female, 31-50 | 31.7 (8.9) |
| Male, 51-70 | 29.8 (6.2) |
| Female, 51-70 | 31.6 (8.4) |
| Male, 71+ | 28.8 (5.2) |
| Female, 71+ | 29.4 (6.4) |
| **Race/ethnicity, % (n)** |  |
| Mexican American | 12% (886) |
| Non-Hispanic Asian | 11% (819) |
| Non-Hispanic Black | 27% (2,007) |
| Non-Hispanic White | 36% (2,724) |
| Other Hispanic | 10% (770) |
| Other Race - Including Mutli-Racial | 4.8% (366) |
| **Education, % (n)** |  |
| Less than 9th grade | 7% (504) |
| 9-11th grade (Includes 12th grade with no diploma) | 11% (784) |
| High school graduate/GED or equivalent | 24% (1,785) |
| Some college or AA degree | 33% (2,469) |
| College graduate or above | 25% (1,854) |
| **Marital status, % (n)** |  |
| Married/Living with Partner | 58% (4,293) |
| Never married | 19% (1,428) |
| Widowed/Divorced/Separated | 23% (1,676) |
| **Poverty-income ratio, % (n)** |  |
| <185% | 37% (2,783) |
| 185-399% | 26% (1,996) |
| ≥400% | 24% (1,847) |
| Missing | 12% (946) |
| *^1^* % (n); Mean (SD) | |

^*^ Data from the National Health and Nutrition Examination Survey (NHANES) 2017-2020[1].

## **S2 Table.** Baseline and counterfactual scenarios: US adults’ (≥19 y) usual mean energy and targeted nutrient intakes by DRI age/sex group (n=7,572)

| **S2.1 Table.** Potential changes in usual energy intake of implementing a mandatory nutrient-specific FOPL in the US (≥19 y), overall and by DRI age/sex group. Counterfactual energy intake considering reductions of calorie contribution from changes observed in sugar and saturated fat. | | | | | | | | | |
| --- | --- | --- | --- | --- | --- | --- | --- | --- | --- |
| ***Energy (kcal/d)*** | | | | | | | | | |
|  | ***n*** | ***Baseline mean*** | *SE* | ***S1 mean*** | *SE* | *S1 ∆* | ***S2 mean*** | *SE* | *S2 ∆* |
| ***Total 19+*** | ***7572*** | ***2077*** | *17* | ***2040*** | *15* | ***38*** | ***2020*** | *14* | ***57*** |
| ***Males*** | | | | | | | | | |
| 19-30 y | 665 | **2440** | 32 | **2399** | 29 | **41** | **2376** | **29** | **64** |
| 31-50 y | 1143 | **2393** | 28 | **2349** | 27 | **44** | **2327** | **27** | **66** |
| 51-70 y | 1353 | **2336** | 32 | **2288** | 28 | **48** | **2266** | **28** | **70** |
| > 71 y | 569 | **2300** | 36 | **2240** | 34 | **60** | **2218** | **34** | **82** |
| ***Females*** | | | | | | | | | |
| 19-30 y | 672 | **1845** | 24 | **1816** | 21 | **29** | **1798** | **21** | **46** |
| 31-50 y | 1220 | **1812** | 17 | **1784** | 14 | **28** | **1767** | **14** | **45** |
| 51-70 y | 1420 | **1769** | 19 | **1739** | 17 | **29** | **1722** | **17** | **46** |
| > 71 y | 530 | **1731** | 27 | **1697** | 25 | **35** | **1680** | **24** | **51** |

Baseline energy and nutrient intakes were estimated using NHANES 2017-2020[1]. Usual intakes were estimated using the National Cancer Institute (NCI) method[2], and analyses were adjusted for age, sex, dietary misreporting status, weekend/weekday, and sequence of dietary recall. Baseline and counterfactual scenarios are described in methods. Counterfactual scenario 1 was based on Taillie, et. al.[3]. Counterfactual scenario 2 was based on Song, et. al.[4]. **SCENARIO 1**, overall changes: sodium (mg) -4.7%; sugars (g) -10.2%; saturated fat (g) -3.9%. **SCENARIO 2**, overall changes: sodium (mg) -7.8%; sugars (g) -7.3%; saturated fat (g) -16.3%. Abbreviations: d=day; DRI=Dietary Reference Intakes; Kcal=kilocalorie; FOPL=front-of-pack labeling; SE=standard error; S1=scenario 1; S2=scenario 2.

| **S2.2 Table.** Potential changes in usual sodium intake of implementing a mandatory nutrient-specific FOPL in the US (≥19 y), overall and by DRI age/sex group. | | | | | | | | | |
| --- | --- | --- | --- | --- | --- | --- | --- | --- | --- |
| ***Sodium (mg/d)*** | | | | | | | | | |
|  | ***n*** | ***Baseline mean*** | *SE* | ***S1 mean*** | *SE* | *S1 ∆* | ***S2 mean*** | *SE* | *S2 ∆* |
| ***Total 19+*** | ***7572*** | ***3349*** | *27* | ***3192**** | *26* | ***156*** | ***3089**** | *25* | ***259*** |
| ***Males*** | | | | | | | | | |
| 19-30 y | 665 | **4038** | 48 | **3850** | 45 | **188** | **3725*** | 44 | **312** |
| 31-50 y | 1143 | **3897** | 33 | **3715*** | 31 | **182** | **3595*** | 30 | **302** |
| 51-70 y | 1353 | **3726** | 42 | **3552*** | 40 | **174** | **3437*** | 39 | **289** |
| > 71 y | 569 | **3608** | 55 | **3439** | 52 | **169** | **3328*** | 50 | **280** |
| ***Females*** | | | | | | | | | |
| 19-30 y | 672 | **3042** | 43 | **2900** | 41 | **142** | **2807*** | 40 | **235** |
| 31-50 y | 1220 | **2936** | 36 | **2799** | 34 | **137** | **2708*** | 33 | **227** |
| 51-70 y | 1420 | **2802** | 40 | **2671** | 38 | **131** | **2585*** | 37 | **217** |
| > 71 y | 530 | **2692** | 55 | **2566** | 52 | **126** | **2483** | 51 | **209** |

Baseline energy and nutrient intakes were estimated using NHANES 2017-2020[1]. Usual intakes were estimated using the National Cancer Institute (NCI) method[2], and analyses were adjusted for age, sex, dietary misreporting status, weekend/weekday, and sequence of dietary recall. Baseline and counterfactual scenarios are described in methods. Counterfactual scenario 1 was based on Taillie, et. al.[3]. Counterfactual scenario 2 was based on Song, et. al.[4]. **SCENARIO 1**, overall changes: sodium (mg) -4.7%; sugars (g) -10.2%; saturated fat (g) -3.9%. **SCENARIO 2**, overall changes: sodium (mg) -7.8%; sugars (g) -7.3%; saturated fat (g) -16.3%. *Indicates a statistically significant difference between baseline mean intakes and counterfactual mean intakes. Abbreviations: d=day; DRI=Dietary Reference Intakes; mg=milligrams; FOPL=front-of-pack labeling; SE=standard error; S1=scenario 1; S2=scenario 2.

| **S2.3 Table.** Potential changes in usual sugar intake of implementing a mandatory nutrient-specific FOPL in the US (≥19 y), overall and by DRI age/sex group. | | | | | | | | | |
| --- | --- | --- | --- | --- | --- | --- | --- | --- | --- |
| ***Sugars (g/d)*** | | | | | | | | | |
|  | ***n*** | ***Baseline mean*** | *SE* | ***S1 mean*** | *SE* | *S1 ∆* | ***S2 mean*** | *SE* | *S2 ∆* |
| ***Total 19+*** | ***7572*** | ***101.1*** | *1.55* | ***91.0**** | *1.39* | ***10.06*** | ***93.9**** | *1.43* | ***7.20*** |
| ***Males*** | | | | | | | | | |
| 19-30 y | 665 | **110.9** | 3.29 | **99.9** | 2.95 | **11.04** | **103.1** | 3.05 | **7.90** |
| 31-50 y | 1143 | **111.3** | 2.92 | **100.2** | 2.62 | **11.08** | **103.3** | 2.71 | **7.93** |
| 51-70 y | 1353 | **111.7** | 2.96 | **100.6** | 2.66 | **11.13** | **103.7** | 2.75 | **7.97** |
| > 71 y | 569 | **112.8** | 3.07 | **101.5** | 2.76 | **11.25** | **104.7** | 2.85 | **8.05** |
| ***Females*** | | | | | | | | | |
| 19-30 y | 672 | **90.0** | 2.10 | **81.1*** | 1.89 | **8.93** | **83.6** | 1.96 | **6.39** |
| 31-50 y | 1220 | **90.8** | 1.19 | **81.8*** | 1.08 | **9.01** | **84.3** | 1.12 | **6.45** |
| 51-70 y | 1420 | **91.6** | 1.34 | **82.5*** | 1.20 | **9.10** | **85.1*** | 1.24 | **6.51** |
| > 71 y | 530 | **92.1** | 1.68 | **82.9*** | 1.51 | **9.16** | **85.5*** | 1.56 | **6.55** |

Baseline energy and nutrient intakes were estimated using NHANES 2017-2020[1]. Usual intakes were estimated using the National Cancer Institute (NCI) method[2], and analyses were adjusted for age, sex, dietary misreporting status, weekend/weekday, and sequence of dietary recall. Baseline and counterfactual scenarios are described in methods. Counterfactual scenario 1 was based on Taillie, et. al.[3]. Counterfactual scenario 2 was based on Song, et. al.[4]. **SCENARIO 1**, overall changes: sodium (mg) -4.7%; sugars (g) -10.2%; saturated fat (g) -3.9%. **SCENARIO 2**, overall changes: sodium (mg) -7.8%; sugars (g) -7.3%; saturated fat (g) -16.3%. *Indicates a statistically significant difference between baseline mean intakes and counterfactual mean intakes. Abbreviations: d=day; DRI=Dietary Reference Intakes; g=grams; FOPL=front-of-pack labeling; SE=standard error; S1=scenario 1; S2=scenario 2.

| **S2.4 Table.** Potential changes in usual saturated fat intake of implementing a mandatory nutrient-specific FOPL in the US (≥19 y), overall and by DRI age/sex group. | | | | | | | | | |
| --- | --- | --- | --- | --- | --- | --- | --- | --- | --- |
| ***Saturated fat (g/d)*** | | | | | | | | | |
|  | ***n*** | ***Baseline mean*** | *SE* | ***S1 mean*** | *SE* | *S1 ∆* | ***S2 mean*** | *SE* | *S2 ∆* |
| ***Total 19+*** | ***7572*** | ***27.6*** | *0.35* | ***26.6*** | *0.34* | ***1.08*** | ***23.1**** | *0.29* | ***4.49*** |
| ***Males*** | | | | | | | | | |
| 19-30 y | 665 | **31.4** | 0.45 | **30.2** | 0.44 | **1.22** | **26.3*** | 0.38 | **5.12** |
| 31-50 y | 1143 | **31.2** | 0.45 | **30.0** | 0.43 | **1.22** | **26.1*** | 0.37 | **5.08** |
| 51-70 y | 1353 | **30.9** | 0.58 | **29.8** | 0.56 | **1.21** | **25.9*** | 0.48 | **5.04** |
| > 71 y | 569 | **30.9** | 0.73 | **29.7** | 0.70 | **1.20** | **25.9*** | 0.61 | **5.03** |
| ***Females*** | | | | | | | | | |
| 19-30 y | 672 | **24.4** | 0.34 | **23.4** | 0.33 | **0.95** | **20.4*** | 0.29 | **3.96** |
| 31-50 y | 1220 | **24.3** | 0.33 | **23.4** | 0.32 | **0.95** | **20.4*** | 0.28 | **3.95** |
| 51-70 y | 1420 | **24.2** | 0.48 | **23.2** | 0.47 | **0.94** | **20.3*** | 0.40 | **3.93** |
| > 71 y | 530 | **24.0** | 0.67 | **23.1** | 0.64 | **0.93** | **20.1*** | 0.56 | **3.91** |

Baseline energy and nutrient intakes were estimated using NHANES 2017-2020[1]. Usual intakes were estimated using the National Cancer Institute (NCI) method[2], and analyses were adjusted for age, sex, dietary misreporting status, weekend/weekday, and sequence of dietary recall. Baseline and counterfactual scenarios are described in methods. Counterfactual scenario 1 was based on Taillie, et. al.[3]. Counterfactual scenario 2 was based on Song, et. al.[4]. **SCENARIO 1**, overall changes: sodium (mg) -4.7%; sugars (g) -10.2%; saturated fat (g) -3.9%. **SCENARIO 2**, overall changes: sodium (mg) -7.8%; sugars (g) -7.3%; saturated fat (g) -16.3%. *Indicates a statistically significant difference between baseline mean intakes and counterfactual mean intakes. Abbreviations: d=day; DRI=Dietary Reference Intakes; g=grams; FOPL=front-of-pack labeling; SE=standard error; S1=scenario 1; S2=scenario 2.

| **S2.5 Table.** Potential changes in percentage of total energy from saturated fat intake of implementing a mandatory nutrient-specific FOPL in the US (≥19 y), overall and by DRI age/sex group. | | | | | | | | | |
| --- | --- | --- | --- | --- | --- | --- | --- | --- | --- |
| ***Saturated fat (% TE/d)*** | | | | | | | | | |
|  | ***n*** | ***Baseline mean*** | *SE* | ***S1 mean*** | *SE* | *S1 ∆* | ***S2 mean*** | *SE* | *S2 ∆* |
| ***Total 19+*** | ***7572*** | ***11.76*** | *0.12* | ***11.70*** | *0.12* | ***0.06*** | ***11.25**** | *0.12* | ***0.52*** |
| ***Males*** | | | | | | | | | |
| 19-30 y | 665 | **11.42** | 0.11 | **11.36** | 0.11 | **0.07** | **10.91*** | 0.10 | **0.51** |
| 31-50 y | 1143 | **11.57** | 0.09 | **11.51** | 0.09 | **0.07** | **11.05*** | 0.09 | **0.52** |
| 51-70 y | 1353 | **11.77** | 0.13 | **11.70** | 0.13 | **0.07** | **11.24** | 0.13 | **0.52** |
| > 71 y | 569 | **11.94** | 0.20 | **11.88** | 0.20 | **0.07** | **11.41** | 0.19 | **0.53** |
| ***Females*** | | | | | | | | | |
| 19-30 y | 672 | **11.61** | 0.12 | **11.55** | 0.12 | **0.06** | **11.11*** | 0.12 | **0.50** |
| 31-50 y | 1220 | **11.79** | 0.15 | **11.73** | 0.15 | **0.06** | **11.28** | 0.14 | **0.51** |
| 51-70 y | 1420 | **12.00** | 0.21 | **11.94** | 0.21 | **0.06** | **11.48** | 0.21 | **0.51** |
| > 71 y | 530 | **12.16** | 0.28 | **12.10** | 0.28 | **0.06** | **11.64** | 0.28 | **0.52** |

Baseline energy and nutrient intakes were estimated using NHANES 2017-2020[1]. Usual intakes were estimated using the National Cancer Institute (NCI) method[2], and analyses were adjusted for age, sex, dietary misreporting status, weekend/weekday, and sequence of dietary recall. Baseline and counterfactual scenarios are described in methods. Counterfactual scenario 1 was based on Taillie, et. al.[3]. Counterfactual scenario 2 was based on Song, et. al.[4]. **SCENARIO 1**, overall changes: sodium (mg) -4.7%; sugars (g) -10.2%; saturated fat (g) -3.9%. **SCENARIO 2**, overall changes: sodium (mg) -7.8%; sugars (g) -7.3%; saturated fat (g) -16.3%. *Indicates a statistically significant difference between baseline mean intakes and counterfactual mean intakes. Abbreviations: d=day; DRI=Dietary Reference Intakes; TE=total energy; FOPL=front-of-pack labeling; SE=standard error; S1=scenario 1; S2=scenario 2.

| **S2.6 Table.** Potential changes in usual sugar intake of implementing a mandatory nutrient-specific FOPL in the US (≥19 y), overall and by DRI age/sex group. | | | | | | | | | |
| --- | --- | --- | --- | --- | --- | --- | --- | --- | --- |
| ***Added sugars (g/d)*** | | | | | | | | | |
|  | ***n*** | ***Baseline mean*** | *SE* | ***S1 mean*** | *SE* | *S1 ∆* | ***S2 mean*** | *SE* | *S2 ∆* |
| ***Total 19+*** | ***7572*** | ***66.8*** | *1.73* | ***60.1**** | *1.55* | ***6.70*** | ***62.0*** | *1.60* | ***4.79*** |
| ***Males*** | | | | | | | | | |
| 19-30 y | 665 | **79.1** | 2.92 | **71.2** | 2.62 | **7.88** | **73.4** | 2.70 | **5.64** |
| 31-50 y | 1143 | **76.0** | 2.59 | **68.4** | 2.33 | **7.59** | **70.6** | 2.40 | **5.43** |
| 51-70 y | 1353 | **72.3** | 2.41 | **65.1** | 2.17 | **7.24** | **67.2** | 2.24 | **5.18** |
| > 71 y | 569 | **69.4** | 2.66 | **62.5** | 2.39 | **6.96** | **64.5** | 2.47 | **4.98** |
| ***Females*** | | | | | | | | | |
| 19-30 y | 672 | **62.4** | 2.33 | **56.1** | 2.10 | **6.27** | **57.9** | 2.16 | **4.48** |
| 31-50 y | 1220 | **60.5** | 1.69 | **54.4** | 1.51 | **6.09** | **56.1** | 1.56 | **4.36** |
| 51-70 y | 1420 | **57.6** | 1.87 | **51.8** | 1.68 | **5.81** | **53.5** | 1.73 | **4.16** |
| > 71 y | 530 | **54.8** | 2.04 | **49.3** | 1.83 | **5.54** | **50.9** | 1.89 | **3.97** |

Baseline energy and nutrient intakes were estimated using NHANES 2017-2020[1]. Usual intakes were estimated using the National Cancer Institute (NCI) method[2], and analyses were adjusted for age, sex, dietary misreporting status, weekend/weekday, and sequence of dietary recall. Baseline and counterfactual scenarios are described in methods. Counterfactual scenario 1 was based on Taillie, et. al.[3]. Counterfactual scenario 2 was based on Song, et. al.[4]. **SCENARIO 1**, overall changes: sodium (mg) -4.7%; sugars (g) -10.2%; saturated fat (g) -3.9%. **SCENARIO 2**, overall changes: sodium (mg) -7.8%; sugars (g) -7.3%; saturated fat (g) -16.3%. *Indicates a statistically significant difference between baseline mean intakes and counterfactual mean intakes. Abbreviations: d=day; DRI=Dietary Reference Intakes; g=grams; FOPL=front-of-pack labeling; SE=standard error; S1=scenario 1; S2=scenario 2.

## **S3 Table.** Diet related NCD deaths that could be averted or delayed in the US due to changes in calorie and nutrients-of-concern content in food and beverage purchases in the presence of nutrient-specific ‘high in’ FOPL.

| **Sensitivity analysis scenario 1**: Estimated number of deaths that could be averted or delayed in the US after modeling reductions of targeted nutrients intakes based on Chilean ‘high in’ FOPL evaluations (overall changes: sodium -4.7%, total sugars -10.2%, sat fat -3.9%) - by cause of death (95% UI). **Changes applied to added sugars.** | | | | | | | | | | | |
| --- | --- | --- | --- | --- | --- | --- | --- | --- | --- | --- | --- |
| **Cause of death (ICD-10 Code) ^1^** | **Total (mean, 95% UI) ^2^** | | **%** | | **Males (mean, 95% UI) ^2^** | | **%** | | | **Females (mean, 95% UI) ^2^** | **%** |
| **Total deaths averted or delayed** | | **67,113 (60,374, 73,641)** | | **100.0** | | **41,775 (37,719, 45,802)** | **100** | | | **25,204 (22,293, 27,960)** | **100** |
| *Total deaths prevented under 75* | | 26,468 (23,753, 29,225) | | 39.4 | | 19,283 (17,286, 21,274) | 46.2 | | | 7,123 (6,277, 7,934) | 28.3 |
| **Cardiovascular diseases** | | **50,252 (44,081, 56,531)** | | **74.9** | | **31,075 (27,306, 34,840)** | **74.4** | | | **19,067 (16,323, 21,749)** | **75.7** |
| Ischaemic heart disease (I20-25) | | 21,583 (18,945, 24,273) | | 32.2 | | 15,480 (13,355, 17,476) | 37.1 | | | 6,084 (4,889, 7,303) | 24.1 |
| Cerebrovascular disease (I60-69) | | 7,696 (6,133, 9,297) | | 11.5 | | 3,971 (3,158, 4,795) | 9.5 | | | 3,731 (2,963, 4,496) | 14.8 |
| Heart failure (I50) | | 8,326 (5,843, 10,598) | | 12.4 | | 4,533 (3,180, 5,883) | 10.9 | | | 3,716 (2,610, 4,752) | 14.7 |
| Aortic aneurysm (I71) | | 111 (48, 180) | | 0.2 | | 72 (29, 121) | 0.2 | | | 38 (15, 63) | 0.2 |
| Pulmonary embolism (I26) | | 49 (16, 101) | | 0.1 | | 27 (8, 55) | 0.1 | | | 23 (7, 46) | 0.1 |
| Rheumatic heart disease (I05-09) | | 18 (5, 38) | | 0.0 | | 7 (2, 16) | 0.0 | | | 11 (3, 22) | 0.0 |
| Hypertensive disease (I10-15) | | 12,512 (9,909, 14,968) | | 18.6 | | 7,021 (5,607, 8,346) | 16.8 | | | 5,460 (4,329, 6,563) | 21.7 |
| **Diabetes (E11, E14)** | | **8,615 (6,947, 10,075)** | | **12.8** | | **5,595 (4,502, 6,562)** | **13.4** | | | **3,018 (2,448, 3,509)** | **12.0** |
| **Cancer** | | **2,952 (2,233, 3,649)** | | **4.4** | | **1,606 (1,177, 2,025)** | **3.8** | | | **1,348 (1,035, 1,666)** | **5.3** |
| Pancreas (C25) | | 673 (116, 1,209) | | 1.0 | | 409 (83, 737) | 1.0 | | | 261 (42, 478) | 1.0 |
| Colorectum (C18-20) | | 1,172 (787, 1,536) | | 1.7 | | 729 (492, 956) | 1.7 | | | 443 (300, 582) | 1.8 |
| Breast (C50) | | 123 (-29, 278) | | 0.2 | | 0 | 0.0 | | | 123 (-33, 278) | 0.5 |
| Endometrium (C54.1) | | 329 (238, 416) | | 0.5 | | 0 | 0.0 | | | 330 (239, 418) | 1.3 |
| Gallbladder (C23) | | 62 (41, 82) | | 0.1 | | 26 (18, 35) | 0.1 | | | 36 (24, 48) | 0.1 |
| Kidney (C64) | | 598 (478, 720) | | 0.9 | | 440 (350, 530) | 1.1 | | | 157 (123, 188) | 0.6 |
| **Chronic renal failure (N18)** | | **2,360 (1,218, 3,488)** | | **3.5** | | **1,438 (729, 2,109)** | | **3.4** | **932 (484, 1,379)** | | **3.7** |
| **Liver disease (K70, K74)** | | **2,952 (1,987, 3,833)** | | **4.4** | | **2,110 (1,404, 2,755)** | | **5.1** | **836 (559, 1,075)** | | **3.3** |

| **Sensitivity analysis scenario 2**: Estimated number of deaths that could be averted or delayed in the US after modeling reductions of targeted nutrient intakes based on a meta-analysis that looked at the impact of nutrient warning FOPL on changing consumers' food and beverages purchasing behavior (overall changes sodium -7.8%, total sugars -7.3%, sat fat -16.3%) - by cause of death (95% UI). **Changes applied to added sugars.** | | | | | | |
| --- | --- | --- | --- | --- | --- | --- |
| **Cause of death (ICD-10 Code) ^1^** | **Total (mean, 95% UI) ^2^** | **%** | **Males (mean, 95% UI) ^2^** | **%** | **Females (mean, 95% UI) ^2^** | **%** |
| **Total deaths averted or delayed** | **117,888 (107,218, 128,741)** | **100.0** | **68,711 (62,475, 75,060)** | **100** | **49,227 (44,156, 53,989)** | **100** |
| *Total deaths prevented under 75* | 48,090 (43,547, 52,522) | 40.8 | 33,315 (30,182, 36,526) | 48.5 | 14,784 (13,264, 16,203) | 30.0 |
| **Cardiovascular diseases** | **87,962 (77,972, 98,301)** | **74.6** | **51,177 (45,376, 56,997)** | **74.5** | **36,840 (32,108, 41,388)** | **74.8** |
| Ischaemic heart disease (I20-25) | 38,812 (34,407, 43,230) | 32.9 | 26,388 (23,173, 29,494) | 38.4 | 12,440 (10,062, 14,728) | 25.3 |
| Cerebrovascular disease (I60-69) | 13,352 (10,560, 16,086) | 11.3 | 6,303 (4,975, 7,592) | 9.2 | 7,039 (5,597, 8,398) | 14.3 |
| Heart failure (I50) | 14,178 (10,045, 18,214) | 12.0 | 7,131 (5,048, 9,052) | 10.4 | 7,062 (5,038, 8,990) | 14.3 |
| Aortic aneurysm (I71) | 182 (74, 303) | 0.2 | 119 (52, 198) | 0.2 | 62 (26, 103) | 0.1 |
| Pulmonary embolism (I26) | 82 (25, 166) | 0.1 | 45 (14, 89) | 0.1 | 37 (12, 75) | 0.1 |
| Rheumatic heart disease (I05-09) | 30 (8, 62) | 0.0 | 12 (4, 26) | 0.0 | 17 (5, 36) | 0.0 |
| Hypertensive disease (I10-15) | 21,437 (17,191, 25,442) | 18.2 | 11,175 (9,068, 13,275) | 16.3 | 10,241 (8,264, 12,030) | 20.8 |
| **Diabetes (E11, E14)** | **15,072 (12,157, 17,614)** | **12.8** | **9,011 (7,295, 10,591)** | **13.1** | **6,012 (4,880, 6,958)** | **12.2** |
| **Cancer** | **5,502 (4,217, 6,800)** | **4.7** | **2,711 (2,037, 3,405)** | **3.9** | **2,780 (2,141, 3,437)** | **5.6** |
| Pancreas (C25) | 1,241 (274, 2,224) | 1.1 | 698 (161, 1,245) | 1.0 | 558 (121, 1,001) | 1.1 |
| Colorectum (C18-20) | 2,166 (1,465, 2,860) | 1.8 | 1,242 (832, 1,636) | 1.8 | 927 (631, 1,220) | 1.9 |
| Breast (C50) | 215 (-100, 538) | 0.2 | 0 | 0.0 | 213 (-119, 532) | 0.4 |
| Endometrium (C54.1) | 685 (505, 862) | 0.6 | 0 | 0.0 | 687 (499, 862) | 1.4 |
| Gallbladder (C23) | 119 (81, 157) | 0.1 | 44 (30, 58) | 0.1 | 76 (52, 99) | 0.2 |
| Kidney (C64) | 1,063 (846, 1,282) | 0.9 | 741 (588, 892) | 1.1 | 325 (260, 389) | 0.7 |
| **Chronic renal failure (N18)** | **4,178 (2,099, 6,112)** | **3.5** | **2,326 (1,169, 3,387)** | **3.4** | **1,876 (946, 2,738)** | **3.8** |
| **Liver disease (K70, K74)** | **5,288 (3,546, 6,868)** | **4.5** | **3,548 (2,373, 4,637)** | **5.2** | **1,756 (1,181, 2,252)** | **3.6** |

^1^WHO, International Statistical Classification of Diseases and Related Health Problems, Tenth Revision.

^2^95% Uncertainty Interval (UI) are based on 10,000 iterations of Monte Carlo analysis built in PRIME.

Total deaths averted or delayed represent less than the sum of the individual diet related NCD mortality causes given that double counting has been accounted for in PRIME during the modeling process. The same applies to the sum of CVDs and cancers.

Potential diet related NCD deaths that could be averted or delayed were estimated using the PRIME model[5]. Inputs for the model included, 1) population demographics; 2) mortality data associated with diet related NCDs, obtained from the publicly available Centers for Disease Control and Prevention (CDC) WONDER datasets, 2019[6, 7]; and 3) baseline and counterfactual dietary intakes.

**References**

1. Stierman B, Afful J, Carroll MD, Chen T-C, Davy O, Fink S, et al. National Health and Nutrition Examination Survey 2017–March 2020 prepandemic data files development of files and prevalence estimates for selected health outcomes. 2021.

2. National Cancer Institute. Usual Dietary Intakes: SAS Macros for the NCI Method 2018 [cited 2024 September 20]. Available from: <https://epi.grants.cancer.gov/diet/usualintakes/macros.html>.

3. Taillie LS, Bercholz M, Popkin B, Reyes M, Colchero MA, Corvalán C. Changes in food purchases after the Chilean policies on food labelling, marketing, and sales in schools: a before and after study. The Lancet Planetary Health. 2021;5(8):e526-e33.

4. Song J, Brown MK, Tan M, MacGregor GA, Webster J, Campbell NR, et al. Impact of color-coded and warning nutrition labelling schemes: A systematic review and network meta-analysis. PLoS Medicine. 2021;18(10):e1003765.

5. Scarborough P, Harrington RA, Mizdrak A, Zhou LM, Doherty A. The preventable risk integrated ModEl and its use to estimate the health impact of public health policy scenarios. Scientifica. 2014;2014.

6. Centers for Disease Control and Prevention CDC WONDER. Underlying Cause of Death, 1999-2020 Request. Deaths occurring through 2020 2019 [cited 2024 March 9]. Available from: <https://wonder.cdc.gov/ucd-icd10.html>.

7. Centers for Disease Control and Prevention CDC WONDER. Bridged-Race Population Estimates 1990-2019 Request 2019 [cited 2024 March 9]. Available from: <https://wonder.cdc.gov/bridged-race-v2019.html>.
